# Accessibility, acceptability, and usage during COVID-19 of the evidence-based intervention Healthy Eating and Active Living Taught at Home (HEALTH): implications for implementation strategies

**DOI:** 10.1186/s12966-026-01896-y

**Published:** 2026-02-24

**Authors:** Amanda Gilbert, Debra Haire-Joshu, Alexandra B. Morshed, Cynthia D. Schwarz, Allison Kemner, Rachel G. Tabak

**Affiliations:** 1 Prevention Research Center at Washington University School of Public Health, St. Louis, MO USA; 2 Center for Diabetes Translation Research at Washington University School of Public Health, St. Louis, MO USA; 3 Department of Behavioral, Social, and Health Education Sciences, Rollins School of Public Health, Emory University, Atlanta, GA USA; 4 Parents as Teachers National Center, St. Louis, MO USA

**Keywords:** Implementation, Obesity prevention, Home visiting, Mothers

## Abstract

**Background:**

Healthy Eating and Active Living Taught at Home (HEALTH) is an evidence-based intervention (EBI) embedded in Parents as Teachers (PAT) home visiting to improve weight outcomes among mothers. Public health impact of HEALTH relies on EBI coverage (accessibility, acceptability, usage). Therefore, the purpose of this study is to identify coverage strengths/gaps which can inform dissemination and implementation (D&I) strategies to increase HEALTH coverage.

**Methods:**

We conducted descriptive analyses using baseline data and home visitor documentation of PAT visits from the HEALTH D&I study, which took place during COVID-19. Coverage was measured by expanding on RE-AIM with the adapted Shengelia et al. Access, utilization, quality, and effective coverage framework and included: accessibility (home visitor referrals to HEALTH), acceptability (mother self-report satisfaction), usage (number of visits mothers received from the home visitor, proportion of HEALTH content delivered, proportion of HEALTH lessons delivered).

**Results:**

In the HEALTH D&I study, 67% of home visitors made at least one referral to HEALTH; however, 33% made no HEALTH referrals. The average number of referrals was 2.05 (SD = 2.38). Most mothers (80%) were satisfied with HEALTH. The average number of visits mothers received was 13.91 (SD = 10.62). On average 39% of all 24 HEALTH lessons, 66% of the eight core HEALTH lessons, and 33% of the HEALTH handouts were delivered. Only 26% of mothers received all 8 core HEALTH lessons. These lessons focus on goals and basic information on healthy eating and physical activity (e.g., Your Health Goals, Let’s Get Moving!, Making Healthy Beverage Habits) and make up the core section of the HEALTH curriculum.

**Conclusions:**

This study demonstrates integration of D&I frameworks to evaluate D&I outcomes and offers insights into strategies for EBIs in community settings and insights into service delivery during COVID-19. Findings show strong EBI coverage (acceptability) and gaps (accessibility; usage) supporting the need for implementation strategies to increase adoption (e.g., building partner relationships, choosing strategic partners, ) and support implementation (e.g., facilitate peer learning, create program guide).

**Trial registration:**

NCT03758638 (https//clinicaltrials.gov/study/NCT03758638), registered Nov 29, 2018.

## Introduction

The early years of motherhood are a critical period in life for obesity risk. Weight gain during pregnancy and postpartum weight retention contribute to the risk of developing obesity. Currently, only one in three women meet the Institute of Medicine (IOM) recommended guidelines for gestational weight gain, and women who exceed these recommendations retain on average an extra six to seven pounds one year post-partum [1–3]. Research estimates around 50% of women retain over 10 additional pounds a year after giving birth [4]. In addition to pregnancy, the caregiving and financial demands of parenthood can lead to obesity by limiting time and resources for healthy eating and being active [5]. Stress resulting from these parental responsibilities can also cause weight gain by influencing neurobiological mechanisms (e.g., increased levels of cortisol) and leading to unhealthy behaviors (e.g., eating high calorie and nutrient poor foods) [6–8]. Developing obesity during this period in life increases the risk women will develop other chronic diseases such as cardiovascular disease, diabetes, and cancer [9–14]; can negatively affect pregnancy outcomes [1, 2, 15–19]; and increases the risk children and future generations develop obesity [17].

Healthy Eating and Active Living Taught at Home (HEALTH) is an evidence-based intervention (EBI) tested through high quality randomized control trials [20–24], that promotes healthy living and weight gain prevention among mothers. HEALTH is a 24-lesson curriculum adapted from the diabetes prevention program and delivered through the Parents as Teachers (PAT) home visiting program [20–24]. Around 2,300 PAT sites serve 186,499 families, reaching 222,549 children across all 50 states [25]. Through PAT, home visitors (providers hired from the communities they serve) conduct personal visits with families beginning during pregnancy and until the youngest child in the home enters kindergarten, to provide education, support, and referrals for child development and family wellbeing [26]. Previous research has shown HEALTH is effective at reducing obesity risk for mothers with young children, as well as during pregnancy and postpartum. One study found mothers who were overweight or had obesity were more likely to achieve and maintain a 5% weight loss over 24 months than mothers who did not receive HEALTH [20]. This study also showed HEALTH improved health behaviors such as physical activity and nutrition, waist circumference, and blood pressure outcomes [20]. Another study found the intervention was effective at preventing excess gestational weight gain, post-partum weight retention, and in promoting health during the perinatal period [22–24].

Though HEALTH is effective, its public health impact requires large scale participation. We posit that HEALTH is widely available because it is delivered through PAT which has significant reach among mothers; however, it is unclear the extent to which HEALTH is accessible, acceptable, and usable. Evidence relating to dissemination and implementation of EBIs in community settings (e.g., social service organizations, schools, workplaces) is less established than dissemination and implementation of EBIs in clinical settings [27–30]. This gap impedes translating evidence into practice in community settings, which experience different implementation determinants than EBIs in clinical settings [31–33]. Community settings delivering EBIs have the potential to reach populations, since they exist within the communities they serve [34, 35]. Limited knowledge around dissemination and implementation in community settings therefore influences the potential for EBIs to reach people and thus ensure they can benefit from the EBI.

This study analyzed data from the HEALTH Dissemination and Implementation study (HEALTH D&I) which took place from 2019 to 2025, during the height of the COVID-19 pandemic. The timing of this study coincided with increased stressors for mothers and families [36, 37], adjustments to home visiting and PAT service delivery (virtual visits and phone calls/text between visits instead of in-person visits) [38], and importance of home visiting and community-based organizations to meet the needs of mothers and families [39, 40]. Describing implementation of an intervention embedded in home visiting during the COVID-19 pandemic can provide insights into home visiting resilience and flexibility for reaching mothers and delivering EBIs during external stressors. The aims of this study are thus to (1) describe indicators of HEALTH implementation during the pandemic and (2) identify opportunities to support HEALTH coverage (i.e., accessibility, acceptability, and usability) during dissemination and implementation.

## Methods

### Study design

This study is an analysis of implementation-related data from the HEALTH D&I study. HEALTH D&I is a pragmatic hybrid 2 cluster-randomized trial testing the dissemination and implementation of the HEALTH intervention as part of PAT [41]. Recruitment for the HEALTH D&I study occurred from March, 2019-June, 2022 and intervention delivery occurred from March, 2019-July, 2024. A detailed description of HEALTH D&I methods is available through the protocol paper [41]. Participants in the study came from 35 PAT sites across 20 states. Randomization occurred at the site level. Sites randomized to the intervention arm received the implementation strategies, specified according to the Expert Recommendations for Implementing Change (ERIC) project compilation (ERIC strategy names are in *italics*): the HEALTH intervention materials to deliver to families and training materials (*develop and distribute educational materials*), interactive training through a synchronous web-based experience (consistent with PAT practice; *make training dynamic*) and ongoing technical support (*provide ongoing consultation*) [30]. All intervention sites received the same implementation strategies; sites randomized to the usual care arm did not receive the implementation strategies or access to the HEALTH materials. Strategies were evaluated through both qualitative and quantitative methods [41]. Main outcomes at the mother level of the HEALTH D&I trial were maternal weight, dietary intake, and physical activity [41]. Implementation outcomes were assessed at the home visitor and supervisor level [41]. This study includes only participants at sites randomized to the intervention (HEALTH) arm.

The HEALTH curriculum contains 24 lessons with resources and associated handouts to be delivered by home visitors to mothers during home visits. The curriculum is structured, with eight core lessons and another 16 lessons on health-related topics (e.g., physical activity, nutrition), however, it is meant to be flexible for home visitors to work with mothers to identify lessons with the best fit. The eight core lessons are meant to introduce healthy eating and active living concepts (e.g., the connection between physical activity and health; effect of sugary drinks on health), build healthy living skills (e.g., reading food labels, meal planning, coping with triggers, identifying barriers to being active and ways to address these barriers, self-monitoring), and help mothers set healthy living goals. These eight core lessons are the most intense and structured part of the curriculum. The following lessons (9–24) are focused more on maintaining health behavior changes and monitoring progress. The 24 lessons target different aspects of healthy living.

- One introductory lesson
- Three physical activity lessons (e.g., Being active: A way of Life, Find time for fitness)
- Eleven nutrition lessons (e.g., Healthy beverages, Portion size, Healthy snacking)
- Four behavior change strategies lessons (e.g., You can manage stress, Stay motivated)
- Five executive skills lessons (e.g., Healthy routines, Self-monitoring, Problem solving, Get support)

### Data collection

Data were collected through self-report surveys administered by telephone, iPad, and in person, and through objective measurement (Table 1). Mothers participating in HEALTH D&I completed surveys at baseline, 12- and 24-months. Surveys included demographics, health behaviors, and experience with HEALTH. Home visitors, who make home visits through PAT and delivered the HEALTH intervention, completed surveys before training, after training and at the end of the study on demographics, implementation determinants and outcomes, and experience delivering HEALTH. Home visitors also completed brief surveys (visit records) about HEALTH delivery, after each home visit. The study was approved by the Washington University in St. Louis institutional review board.

**Table 1:** Measurement for HEALTH D&I coverage analysis

Construct	Definition	Data	Operationalization/Example Questions
Coverage			
Accessibility	Whether mothers are aware of and able to participate in HEALTH.	HEALTH D&I study tracking	Number of home visitor referrals to the HEALTH D&I study.
Acceptability	Whether mothers are willing to participate.	12- and 24-month survey	Advice was helpful; information was relevant; HEALTH helped with health behaviors; HEALTH handouts were helpful.^a^
Usage	How much of HEALTH mother’s use.	Visit records	Number of visits mothers received from the home visitor; HEALTH lessons and handouts delivered; Percentage of HEALTH content delivered during each visit.
Home visitor and Mother demographics			
Ethnicity	Self-identified ethnicity.	Baseline survey	What ethnicity do you identify with?
Education	Level of educational attainment.	Baseline survey	What is the highest grade of school that you completed?
Body Mass Index	Whether a mother or home visitor had a healthy weight, was overweight, or had obesity at baseline.	Objective measure and baseline survey	Continuous and calculated using objectively measured weight with self-reported height. Formula: weight in kilograms (kg) divided by height in meters, squared (m^2^)
Home visitor specific demographics			
Years at PAT site	Number of years at PAT site.	Baseline survey	How many years have you been working at your site?
Years in PAT position	Number of years in PAT position.	Baseline survey	How many years have you been working at your current position?
Implementation intentions/determinants^b^			
* Self-efficacy*	Intention to adopt HEALTH based on home visitor’s sense of self-efficacy as it relates to themselves and their site for delivering HEALTH.	Baseline survey	Our site has the expertise needed in order to implement HEALTH as prescribed; I am confident that I can implement HEALTH as prescribed at our site.^c^
* Knowledge and beliefs about HEALTH*	Intention to adopt HEALTH based on home visitor knowledge and beliefs of HEALTH.	Baseline survey	I am aware of curricula which address healthy weight; I know the status of healthy weight education in my site.^d^
* Appeal of HEALTH*	Intention to adopt HEALTH based on home visitors perceptions of HEALTH appeal.	Baseline survey	If you received training in a curriculum that was new to you, how likely would you be to adopt it if it was intuitively appealing.^e^ If you received training in a curriculum that was new to you, how likely would you be to adopt it if it “made sense” to you.^e^
* Openness to HEALTH*	Intention to adopt HEALTH based on home visitors openness to a new curriculum.	Baseline survey	I like to use a new curriculum to help families; I am willing to try a new curriculum even if I have to follow a manual.^e^
Mother specific demographics and health behaviors			
Income	Annual household income.	Baseline survey	What is your annual household income from all sources?
SNAP/WIC	Participation in WIC or SNAP	Baseline survey	Do you receive help from any of the following programs?
Food insecurity	Experience of food insecurity	Baseline survey	We worried whether our food would run out before we got money to buy more.^f^ The food that we bought just didn’t last, and we didn’t have money to get more.^f^
Physical activity^g^	Level of physical activity	Baseline survey	During the last 7 days, on how many days did you do vigorous physical activities like heavy lifting, digging, aerobics, or fast bicycling? During the last 7 days, on how many days did you do moderate physical activities like carrying light loads, bicycling at a regular pace, or doubles tennis? Do not include walking.
*Dietary intake* ^h^			
* Added sugar*	Daily tsp of added sugar	Baseline survey	During the past month, how often did you eat doughnuts, sweet rolls, Danish, muffins, pan dulce, or pop-tarts?
* Fruits and vegetables*	Daily cups of fruits and vegetables	Baseline survey	During the past month, how often did you eat fruit? Include fresh, frozen or canned fruit. During the past month, not including what you just told me about (green salads, potatoes, cooked dried beans), how often did you eat other vegetables?
* Fiber*	Daily grams of fiber	Baseline survey	During the past month, how often did you eat whole grain bread including toast, rolls and in sandwiches? Whole grain breads include whole wheat, rye, oatmeal and pumpernickel.

^a^Response options included Strongly agree to Strongly disagree on a 5-point Likert scale.^b^ Constructs informed by implementation science frameworks Reach, Effectiveness, Adoption, Implementation, and Maintenance (RE-AIM) and Consolidated Framework for Implementation Research (CFIR) [1, 2]. ^c^Response options include Strongly agree to Strongly disagree on a 7-point Likert scale. ^d^Response options include Strongly agree to Strongly disagree on a 4-point Likert scale. ^e^Response options included Not at all to A very great extent on a 5-point Likert scale. ^f^Response options included Often true to Never true on a 3-point Likert scale. ^g^Measured using the International Physical Activity Questionnaire (IPAQ) [3]. ^h^Measured using the National Health and Nutrition Examination Survey (NHANES) dietary screening questionnaire [4]

### Measures

#### Coverage

**Fig. 1:**
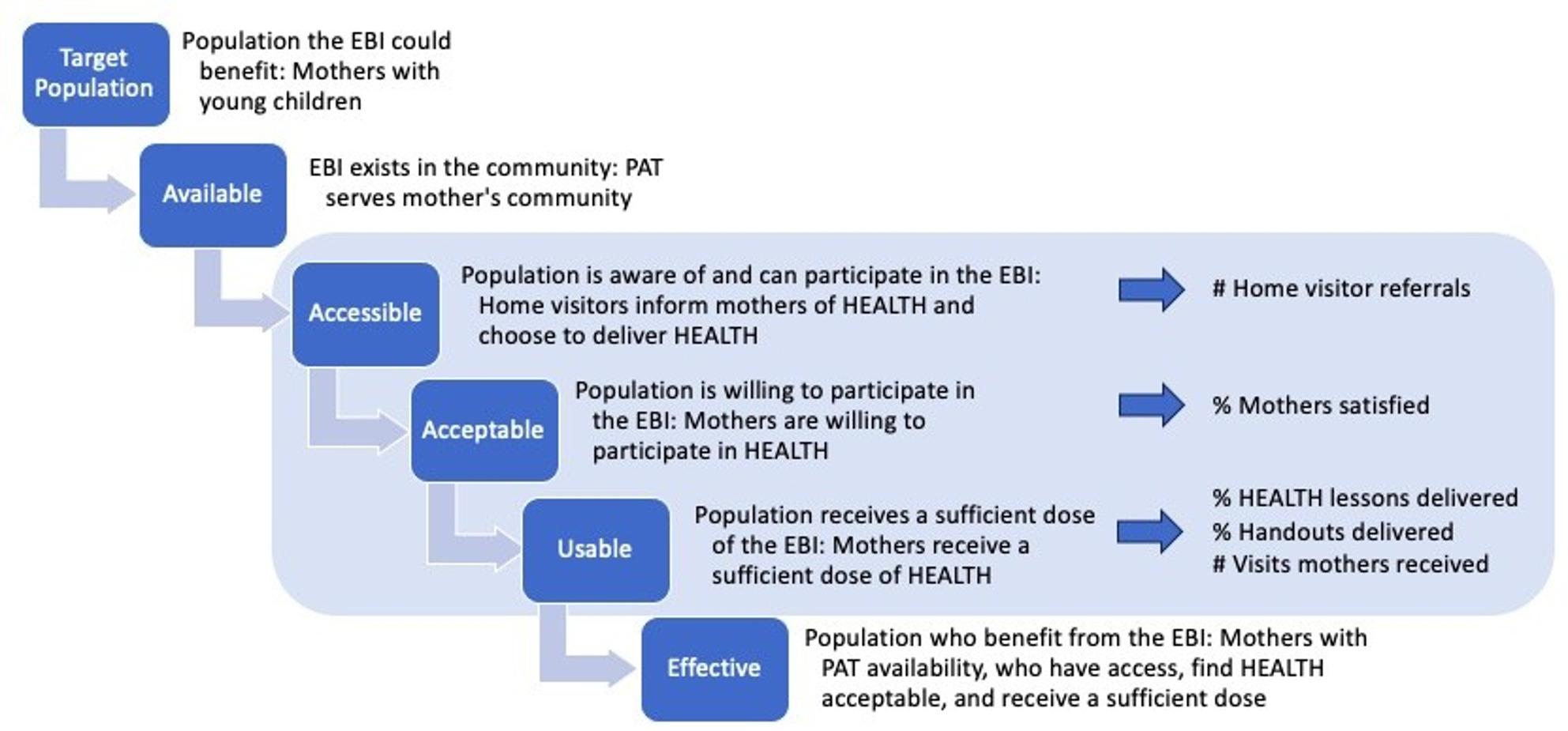
Healthy Eating and Active Living Taught at Home (HEALTH) coverage framework. Adapted from Shengelia et al. 2005 [45]

The HEALTH D&I study was organized around the Reach, Effectiveness, Adoption, Implementation, and Maintenance (RE-AIM) and the Consolidated Framework for Implementation Research (CFIR) frameworks which outline implementation determinants and outcomes [42, 43]. While these frameworks informed quantitative measures for the HEALTH D&I study, ongoing qualitative research activities related to HEALTH implementation with PAT highlighted ways these two frameworks were unable to fully capture reach, adoption, and implementation of HEALTH. RE-AIM constructs have been expanded through integration of other frameworks and models [44]. To expand on these constructs in this study, elements of the Shengelia et al. *Access*,* utilization*,* quality*,* and effective coverage framework* were integrated with RE-AIM [45]. Through this Coverage framework, reach, adoption, and implementation are seen as a function of the program being available, accessible, acceptable, and usable (Fig. 1). Availability relates to whether HEALTH exists in a mother’s community and expands upon RE-AIM reach by going beyond representativeness of the population willing to participate to representativeness of the population able to participate through having PAT in their community. Accessibility relates to RE-AIM adoption and whether mothers are aware of and able to participate in HEALTH through their home visitor’s choice to refer them to the HEALTH program. Accessibility is an outcome (i.e., RE-AIM adoption) and a determinant for RE-AIM reach. Acceptability is whether mothers are satisfied with HEALTH and as such are willing to participate. Acceptability is an implementation outcome, aligns with the CFIR outcomes addendum, and is a determinant for RE-AIM reach and implementation. Acceptability is an antecedent of RE-AIM reach and implementation through participant satisfaction influencing a mother’s willingness to participate (i.e., reach) and thus the extent to which a home visitor can continue to implement and deliver HEALTH (i.e., implementation). Usage captures whether and how much of HEALTH mothers receive. Usage relates to RE-AIM implementation and is a function of fidelity as it represents how closely dose of HEALTH aligns with delivery of recommended core lessons and content topics. Expanding RE-AIM with the coverage framework, provides additional metrics for understanding HEALTH reach, adoption, and implementation and can point to strategies for addressing gaps in effective coverage of HEALTH (the representativeness of and proportion of mothers who can benefit from HEALTH). Using other frameworks combined with RE-AIM can help conceptualize and tease apart the interrelated and overlapping constructs of determinants and outcomes for implementation [44, 46].

HEALTH implementation was measured through expanding RE-AIM reach, adoption, and implementation with elements of the Shengelia et al. Coverage framework [45]. (Fig. 1 and Table 1) *Accessibility* was measured using HEALTH D&I study tracking of home visitor referrals to the HEALTH D&I study. This measure represents a home visitor choosing to deliver HEALTH to the mothers they visit (though not every mother referred to the study was enrolled, as screening for study eligibility was conducted by the study team). *Acceptability* was measured through mother self-reported satisfaction with HEALTH at 12- and 24-months. Mothers were asked to indicate how much they agree with nine items measuring elements of satisfaction with HEALTH (e.g., advice was helpful). For this analysis, these variables were dichotomized into satisfied (strongly agree, agree, neutral) and not satisfied (strongly disagree, disagree). Satisfaction variables at 12- and 24-months were combined into satisfied (satisfied at 12- and 24-months or satisfied at 12-months with missing data at 24-months) and not satisfied (not satisfied at 12- or 24-months or not satisfied at 12 months with missing data at 24 months). Mothers were satisfied with HEALTH if they were satisfied with each of the nine satisfaction items across 12- and 24-months. *Usage* was measured through visit records. Measures include the number of visits mothers received from the home visitor and which HEALTH lessons and handouts were delivered at the visits. Home visitors also indicated whether they talked about the mother’s eating, drinking, or physical activity during the visit (Yes/No) and how much of the HEALTH content was delivered (None, About 25%, About half, About 75%, All of it). For this analysis, we dichotomized how much HEALTH content was delivered into about 50% or more (About half, About 75%, All of it) and less than 50% (None, About 25%).

#### Demographics and health behaviors

Mother and home visitor demographics were measured through self-report surveys and objective measurement. (Table 1) Weight of mothers was measured using a scale, while home visitors self-reported their weight. Body mass index (BMI) was calculated using weight and self-reported height for mothers and educators. Inclusion criteria for mothers in HEALTH D&I was a BMI between 25 and 45 (being overweight or having obesity). *Demographics for mothers and home visitors* included ethnicity (Hispanic/Not Hispanic) and education (High school or less/Some college or technical school/College or more). *Demographics specific to mothers* also included annual household income from all sources (Less than $30,000/$30,00 or more); SNAP or WIC participation (Yes/No); and food insecurity (Yes/No), measured through the Hunger Vital Sign two-item measure [47]. Mothers also self-reported heath behaviors, including physical activity (low/moderate/high) measured with the International Physical Activity Questionnaire [48]; and dietary intake of daily tsp of added sugar, daily cups of fruits and vegetables, and daily grams of fiber (meets guidelines/ does not meet guidelines) using the National Health and Nutrition Examination Survey [49]. *Demographics specific to home visitors* included years at their PAT site; years in PAT position; and information about home visitor level implementation determinants. Implementation determinant measures at the provider level were informed by RE-AIM and CFIR [42, 43]. The specific measures (e.g., self-efficacy; appeal; openness; knowledge, beliefs, and attitudes) were piloted and refined through a small study with PAT supervisors and home visitors [50].

### Data analysis

*Accessibility* was analyzed using a home visitor level dataset which included baseline survey data from home visitors and data on referrals. This dataset included all 171 home visitors who participated in the HEALTH D&I study. Descriptive statistics were calculated to determine the number of referrals to HEALTH made by home visitors and how many referrals each home visitor made. T-tests and chi square analyses were conducted to determine whether differences between home visitors who made at least one referral and those who made no referrals, across demographic and implementation characteristics were statistically significant.

*Acceptability and usage* were analyzed using a mother level dataset, which included baseline, 12- and 24-month survey data reported by the mother and the visit record data completed by the home visitor. When a mother had more than one home visitor during the study period (e.g., turnover of staff within a PAT site), visit record data from the home visitor who provided the most visits for that mother were used. Separate analytic samples were created for acceptability and usage based on available data. Descriptive statistics were used to describe frequency and proportion of mothers who were satisfied, the number of visits mothers received from the home visitor, the HEALTH lessons and handouts delivered, and how much HEALTH content was delivered during each visit. Due to small sample sizes, tests to determine statistical differences between groups (mothers who were satisfied vs. not satisfied; mothers who received all eight core HEALTH lessons vs. mothers who did not) were not conducted.

## Results

*Accessibility* (Table 2): The analytic sample for measuring accessibility included 135 home visitors who completed demographic and implementation surveys out of the 171 home visitors in the study. The average number of referrals made to HEALTH by home visitors was 2.05 (SD = 2.38). Overall, 67.41% (*n* = 91) of home visitors made at least one referral to HEALTH. Among home visitors who made at least one referral, the average number of referrals was 3.04 (SD = 2.31). Home visitors who made at least one referral had a statistically significant (*p* = 0.02) lower BMI (M = 29.36, SD = 0.77) compared to home visitors who made no referrals (M = 32.13, SD = 1.17). While not statistically significant, a higher percentage of home visitors who made at least one referral had a healthy weight (*n* = 28, 30.77%) compared to home visitors who did not make any referrals (*n* = 6, 13.64%). There were trends toward other differences between groups, though these were not statistically significant. Home visitors who made at least one referral tended to have worked at their site longer (M = 5.26, SD = 6.44 years compared to M = 3.77, SD = 4.47 years) and to have worked in PAT longer (M = 4.60, SD = 6.02 years compared to M = 3.36, SD = 3.64 years) than home visitors who made no referrals.

**Table 2:** Description of HEALTH accessibility (*n* = 135)

	Accessibility		
	Made at least one referral to HEALTH (*n* = 91, 67.41%)^a^	Made no referrals to HEALTH (*n* = 44, 32.59%)^a^	*p* value
Number of referrals to HEALTH (M, SD)	3.04 (2.31)	-	
Ethnicity (n,%)			0.39
Hispanic	29 (31.87)	18 (40.91)	
Not Hispanic	60 (65.93)	24 (54.55)	
Unknown	2 (2.20)	2 (4.55 )	
Education (n,%)			0.96
HS or less	5 (5.49)	2 (4.55 )	
Some college/technical school	11 (12.09)	5 (11.36)	
College or more	75 (82.42)	37 (84.09)	
Body Mass Index^b^ (n,%)			0.1
Healthy weight	28 (30.77)	6 (13.64)	
Overweight	27 (29.67)	17 (38.64)	
Obesity	36 (39.56)	21 (47.73)	
Body Mass Index^b^ (M, SD)	29.36 (0.77)	32.13 (1.17)	**0.02**
Years at Site (M, SD)	5.26 (6.44)	3.77 (4.47)	0.92
Years in position (M, SD)	4.60 (6.02)	3.36 (3.64)	0.89
Home visitor level implementation intentions and determinants at baseline (M, SD)			
CFIR^c^ Self-efficacy	5.14 (1.45)	5.56 (1.33)	0.06
CFIR^c^ Knowledge and beliefs about HEALTH	2.80 (0.73)	2.91 (0.70)	0.20
RE-AIM^d^ appeal	3.13 (0.79)	2.82 (0.86)	0.98
RE-AIM^d^ openness	3.19 (0.75)	3.22 (0.76)	0.41

^a^One hundred seventy one home visitors participated in the HEALTH D&I study. Only 135 of 171 home visitors had demographic and implementation data for the analytic sample measuring accessibility. ^b^Body Mass Index calculated using objectively measured weight with self-reported height. Formula: weight in kilograms (kg) divided by height in meters, squared (m^2^). Healthy weight < 25 kg/m^2; Overweight 25 kg/m^2 to 29.9 kg/m^2; Obesity ≥ 30 kg/m^2 [1]. ^c^Measure informed by the Consolidated Framework for Implementation Research (CFIR) [2]. ^d^Measure informed by the Reach, Effectiveness, Adoption, Implementation and Maintenance Framework (RE-AIM) [3]

^*^Accessibility was measured with the home visitor level dataset (baseline survey data from home visitors and study tracking data on referrals)

*Acceptability* (Table 3): The analytic sample for measuring acceptability included 65 mothers who provided demographic data at baseline and acceptability (answered all satisfaction questions) data at 12- and/or 24-months, out of the 166 mothers in the overall sample. The majority, 80% (*n* = 52) of mothers were satisfied with HEALTH. Among mothers who were satisfied with HEALTH, there was trend toward a higher percentage of mothers who identified as Hispanic (*n* = 18, 34.62% compared to *n* = 2, 15.36%), had an educational attainment of a college degree or more (*n* = 21, 40.38% compared to *n* = 0, 0%), and made less than $30,000 in annual household income (*n* = 24, 46.15% compared to *n* = 5, 38.46%). Among mothers who were satisfied with HEALTH, there was a lower percentage of mothers who received WIC or SNAP (*n* = 29, 55.75% compared to *n* = 11, 84.62%) and reported food insecurity (*n* = 12, 23.08% compared to *n* = 7, 53.85%). In the group of mothers who were satisfied with HEALTH, there was a lower percentage of mothers who had obesity at baseline (*n* = 31, 59.62%) compared to mothers who were not satisfied (*n* = 11, 84.62%).

**Table 3:** Description HEALTH acceptability and usage

	Acceptability (*n* = 65)^a^		Usage (*n* = 111)^b^	
	Satisfied^c^ (*n* = 52 80%)	Not satisfied^c^ (*n* = 13, 20%)	Received all 8 core lessons^d^ (*n* = 29, 26.13%)	Did not receive all 8 core lessons^d^ (*n* = 82, 73.87%)
Ethnicity (n,%)				
Hispanic	18 (34.62)	2 (15.38)	8 (27.59)	31 (37.80)
Not Hispanic	33 (63.46)	11 (84.62)	21 (72.41)	48 (58.54)
Unknown	1 (1.92)	0 (0)	0 (0)	3 (3.66)
Education (n,%)				
HS or less	17 (32.69)	7 (53.85)	13 (44.83)	39 (47.56)
Some college/technical school	14 (26.92)	6 (46.15)	8 (27.59)	24 (29.27)
College or more	21(40.38)	0 (0)	8 (27.59)	19 (23.17)
Income (n,%)				
Less than $30,000	24 (46.15)	5 (38.46)	12 (41.38)	44 (53.66)
$30,000 or more	28 (53.85)	8 (61.54)	17 (58.62)	38 (46.34)
SNAP/WIC (n,%)				
Yes	29 (55.75)	11 (84.62)	10 (34.48)	24 (29.27)
No	23 (44.23)	2 (15.38)	19 (65.52)	58 (70.73)
Food insecurity (n,%)				
Yes	12 (23.08)	7 (53.85)	20 (68.97)	59 (71.95)
No	40 (76.92)	6(46.15)	9 (31.03)	23 (28.05)
Physical activity^e^ (n,%)				
Low	29 (55.77)	8 (61.54)	19 (65.52)	53 (64.63)
Medium	14 (26.92)	2 (15.38)	6 (20.69)	15 (18.29)
High	9 (17.31)	3 (23.08)	4 (13.79)	14 (17.07)
Dietary added sugar^f^ (n,%)				
Does not meet guidelines^g^	48 (92.31)	11 (84.62)	26 (89.66)	75 (91.46)
Meets guidelines^g^	4 (7.70)	2 (15.38)	3 (10.34)	7 (8.54)
Dietary fruit and vegetable^f^ (n,%)				
Does not meet guidelines^h^	52 (100)	12 (92.31)	29 (100)	78 (94.12)
Meets guidelines^h^	0 (0)	1 (7.69)	0 (0)	4 (4.88)
Dietary fiber intake^f^ (n,%)				
Does not meet guidelines^i^	51 (98.08)	12 (92.31)	29 (100)	79 (96.34)
Meets guidelines^i^	1 (1.92)	1 (7.69)	0 (0)	3 (3.66)
Body Mass Index^j^ (n,%)				
Overweight	21(40.38)	2 (15.38)	4 (13.79)	32 (39.02)
Obesity	31 (59.62)	11 (84.62)	25 (86.21)	50 (60.98)
Body Mass Index^j^ (M, SD)	32.66 (5.56)	34.82 (5.56)	34.43 (4.67)	33.23 (5.61)

^a^The analytic sample for acceptability includes the 65 mothers who provided demographic data at baseline and acceptability (all satisfaction questions) data at 12- and or 24-months, out of the 166 mothers in the overall sample. ^b^Usage was measured with visit record data, which was completed for 143 mothers. The analytic sample for usage includes the 111 mothers who had complete baseline demographic and visit record data. ^c^Satisfied = Agreed to all 9 satisfaction items; Not satisfied = did not agree to all 9 satisfaction items. ^d^Eight core lessons out of the 24 HEALTH lessons. ^e^Measured using the International Physical Activity Questionnaire [1]. ^f^Measured using National Health and Nutrition Examination Survey (NHANES) [2]. ^g^Daily added sugar (tsp) - Meets guidelines less than 12 tsp [3]. ^h^Daily added fiber (grams) - Meets guidelines 25 g or more [3]. ^i^Daily fruit and vegetables (cups) - Meets guidelines 4 cups or more [3]. ^j^Body Mass Index calculated using objectively measured weight with self-reported height. Formula: weight in kilograms (kg) divided by height in meters, squared (m^2^). Overweight 25 kg/m^2 to 29.9 kg/m^2; Obesity ≥ 30 kg/m^2 [4]

^*^Acceptability and Usage were measured with the mother level dataset (baseline, 12- and 24-month survey data reported by the mother and the visit record data completed by the home visitor)

*Usage* (Tables 3 and 4): The analytic sample for measuring usage included 111 mothers who had complete baseline demographic and visit record data out of the 143 mothers who had visit record data. Table 4 shows that on average, mothers received 13.91 (SD = 10.62) visits from PAT during their time in the study. In terms of the HEALTH content delivered, an average of 39.49% (SD = 27.19) of the 24 HEALTH lessons in the curriculum were delivered. Around 66% (SD = 31.17) of the eight core lessons were delivered. Only 32.5% (SD = 26.84) of the handouts were provided to mothers. Very few mothers received all three physical activity lessons (*n* = 16, 14.41%), all 11 nutrition lessons (*n* = 5, 4.5%), all four behavior change strategy lessons (*n* = 2, 1.8%), and all five executive skills lessons (*n* = 12, 10.8%). Most mothers (*n* = 82, 73.87%) did not receive all eight core lessons (Table 3). For 55.86% (*n* = 62) of mothers, all visits they received contained at least 50% of the HEALTH content for that lesson.

**Table 4:** HEALTH usage (*n* = 111)^a^

**Visits**	
Number of visits mothers received (M, SD)	13.91 (10.62)

**Lessons** ^**b**^	
Proportion of the 24 HEALTH lessons delivered (M, SD)	39.49 (27.19)
Proportion of 3 Physical activity lessons delivered (M, SD)	40.84 (32.31)
Proportion of 11 Nutrition lessons delivered (M, SD)	41.44 (30.86)
Proportion of 4 Behavior change strategies lessons delivered (M, SD)	27.03 (27.41)
Proportion of 5 Executive skill lessons delivered (M, SD)	33.87 (31.66)
Proportion of 8 Core lessons delivered (M, SD)	65.88 (31.17)

**Handouts**	
Proportion of the handouts delivered (M, SD)	32.50 (26.84)

**Mothers (** ***n*** ** = 111)** ^**a**^	
Mothers who received all 3 Physical activity lessons delivered (n,%)	16 (14.41)
Mothers who received all 11 Nutrition lessons delivered (n,%)	5 (4.50)
Mothers who received all 4 Behavior change strategies lessons delivered (n,%)	2 (1.80)
Mothers who received all 5 Executive skill lessons delivered (n,%)	12 (10.81)
Mothers who received all 8 Core lessons delivered (n,%)	29 (26.13)
Mothers where all visits received contained 50% or more of the HEALTH content (n,%)	62 (55.86)
Mothers where at least 80% of visits the parent educator talked about physical activity/nutrition (n.%)	100 (90.09)

^a^Usage was measured with visit record data, which was completed for 143 mothers. The analytic sample for usage includes the 111 mothers who had complete baseline demographic and visit record data. ^b^The 3 physical activity lessons, 11 Nutrition lessons, 4 behavior change strategies lessons, and 5 executive skill lessons do not include the first introductory lesson

Table 3 shows among mothers who received all eight core HEALTH lessons, there was a higher percentage of mothers who identified as not Hispanic (*n* = 21, 72.41% compared to *n* = 48, 58.54%), had obesity (*n* = 25, 86.21% compared to *n* = 50, 60.98%), and made $30,000 or more in annual household income (*n* = 17, 58.62% compared to *n* = 38, 46.34%). Usage did not seem to differ based on food insecurity, receiving WIC/SNAP, physical activity, or dietary intake.

## Discussion

This study addresses gaps in understanding community-based implementation of health-focused EBIs and since the HEALTH D&I study occurred during the COVID-19 pandemic, provides insight into community-based home visiting service delivery during external stressors. Findings from this study indicate mother acceptability of HEALTH was high, with around 80% of mothers reporting satisfaction with the HEALTH curriculum. However, accessibility and usage could be improved. These findings suggest more work could be done to ensure the dissemination and implementation of HEALTH, specifically for increasing access and usage. By expanding the RE-AIM framework with the coverage framework, this study captures additional elements important for assessing implementation of EBIs in community settings.

Results build on previous research suggesting successful dissemination and implementation may require more than several strategies [51]. For example, the HEALTH D&I study employed the implementation strategies of *develop and distribute educational materials*,* make training dynamic*,* provide ongoing technical support*. These strategies likely improved acceptability but were not sufficient on their own or expanded on to the extent necessary to obtain full coverage, especially during COVID-19. The HEALTH D&I trial had rolling recruitment beginning in early 2019 and ran through mid 2022; the intervention was 24-months, such that the intervention continued through mid 2024. The COVID-19 pandemic disrupted recruitment, implementation of HEALTH, and follow up. Many of the study activities (recruitment, data collection), and the remaining baseline, 12- and 24-month follow-up were switched to virtual from in-person. Like other home visiting models, PAT transitioned to providing home visits from in-person to virtual [40, 52]. In the context of COVID-19, these results may also reflect the increased stressors and disruptions for mothers and families [36, 37, 53], which impacted community-based organizations, and specifically home visiting’s ability to reach mothers and deliver EBIs [38–40, 52, 54]. No counterfactual exists to determine what coverage would look like without the impact of COVID-19; however, results still lend insight for opportunities to support implementation in home visiting and community-based settings.

### Accessibility

Referral by parent educators to the HEALTH intervention was a gap identified in this study. While most home visitors (67%) made referrals, and on average home visitors made 2 referrals to HEALTH, 33% of home visitors did not make any referrals. Though comparisons to previous research are limited by the diversity of community settings, types of EBIs, study designs, and implementation outcomes measured [32, 55, 56], HEALTH accessibility seems to align with evidence of adoption in other studies [57]. One community-based study of a physical activity and nutrition intervention delivered through churches found 64% of pastors chose to adopt the intervention [58]. Adoption rates were similar in a review of behavioral interventions, which found provider’s choosing to participate in an intervention was around 79% +/- 28% points [59]. This review was not specific to community settings and the range provided is large. While limitations exist for these comparisons, data from previous research seems to suggest accessibility of HEALTH is like other studies yet still may reflect opportunities for improvement.

It is important to note some limitations regarding measuring accessibility in this study. Because referral to the HEALTH intervention took place as part of a research study, our accessibility measure may be limited due to the HEALTH D&I study criteria (i.e., mothers needed to have a BMI in a certain range and not be pregnant or planning to become pregnant in the next year) rather than an indicator of HEALTH access. These study requirements may have influenced the likelihood a home visitor chose to refer a mother to the HEALTH D&I study to deliver the HEALTH intervention. Further, if a home visitor referred a mother to the HEALTH intervention and they ended up not meeting the screening criteria (screening criteria were assessed by the research team), this may have made home visitors less likely to refer other mothers. High rates of PAT staff turnover occurred during the COVID-19 pandemic, with many home visitors (70, 41%) in the HEALTH D&I study leaving PAT before the HEALTH D&I study ended. This could have influenced how many home visitors made referrals.

Even with these limitations, the data suggests the implementation strategies applied in the current study may not have been adequate to result in uptake and that there may be opportunities for increasing the number of home visitors who opt to use HEALTH with the mothers they visit. The HEALTH D&I study was planned using strategies outlined in the Expert Recommendations for Implementing Change (ERIC) project which was focused primarily on clinical settings [30]. The strategies defined in ERIC may not be specified to implementation in community settings or comprehensive for the unique barriers and opportunities for implementing community based EBI’s. Other strategies identified and refined specifically for community settings may be more effective in addressing coverage gaps [31]. For example, potential strategies for increasing accessibility are either not identified in ERIC (e.g., building partner relationships, choosing strategic partners, and meeting community partner needs) or are identified but could be more fully operationalized for community contexts (e.g., engage potential partners, facilitate peer learning) [30].

Building partner relationships, choosing strategic partners, and meeting community partner needs may be particularly useful in community settings where the health focused EBI may not be the priority or primary mission of the community organization [32], and thus less a priority for providers to adopt and implement. These strategies can help providers meet family social needs through referrals to available resources and shared expertise, freeing up time and attention for providers to choose to learn and deliver other content such as HEALTH [31]. These strategies may be even more important when social needs are high, as was the case for mothers and families during COVID-19 [36, 37, 53]. Engaging potential partners and facilitating peer learning can increase awareness of the EBI and promote dissemination within a community organization and adoption by providers. Since community organizations are embedded within the communities they serve, these strategies can go beyond traditional educational outreach. In community contexts, these strategies can leverage existing community networks and relationships to share information about the EBI and provide opportunities for implementers to learn from each other [31]. These strategies may help address trends identified in this study which suggest less referrals were made by home visitors with less time in their role or at PAT.

### Acceptability

The high HEALTH acceptability among mothers who used HEALTH indicates opportunities for building acceptability coverage of the HEALTH intervention. Most mothers (80%) reported satisfaction with HEALTH. This high level of satisfaction is particularly noteworthy given the high criteria for measuring satisfaction (mothers satisfied for all nine satisfaction questions). It is important to note, since HEALTH is embedded within PAT curricula, it is possible mothers may not be able to distinguish usual PAT content from the HEALTH intervention. These results indicate that if mothers are offered HEALTH and it is accessible to them, they may be likely to find the HEALTH curriculum helpful and useful. They may also be more likely to continue to engage with the HEALTH curriculum over time. Previous research has found significant challenges in ensuring participants remain in a program [60, 61]. HEALTH delivery is designed to fit with how PAT delivers content, which is to allow home visitors to use their professional judgment about what to provide to mothers. This model and the focus on home visitor professional judgement helps ensure mothers get what they need, potentially improving HEALTH acceptability. The HEALTH D&I study trialed implementation strategies such as *make training dynamic*, which may have supported home visitors to use their expertise and relationships with families to deliver HEALTH content in a way that most fit with a mother’s strengths and needs. These findings support the potential of HEALTH to engage mothers over time and to provide education on healthy eating and physical activity.

The group of mothers who were satisfied with HEALTH had higher proportions of mothers who identified as Hispanic and low-income than the group of mothers who did not find HEALTH satisfactory, suggesting HEALTH may be a good fit for and able to engage these groups of mothers. However, more mothers who were not satisfied with HEALTH used WIC/SNAP and reported food insecurity, suggesting HEALTH may not be providing healthy eating content in line with experiences related to food insecurity and use of WIC/SNAP. These gaps in acceptability point to the need for more support for home visitors to navigate adapting the program.

Implementation strategies including those outlined in ISAC such as developing adaptable programs, creating a program guide, and changing adaptable program components can ensure providers adapt EBI content to fit participants who have different backgrounds and experiences, while maintaining program fidelity [30, 31]. Additionally, implementation strategies specific to community settings such as choosing strategic partner organizations, can help providers meet participant social needs by connecting them to available resources [31]. Addressing social needs like food insecurity may make it possible for home visitors to focus on HEALTH content and ensure HEALTH content is relevant to mother’s experiences. These strategies may be particularly important during time such as COVID-19 when social needs like food insecurity are high, and additional resources are needed to alleviate these needs to focus on HEALTH [36, 37, 53].

### Usage

A key finding in this study was the limited usage of HEALTH among mothers who were referred to HEALTH. On average, only 35% of the 24 HEALTH lessons were delivered and only 33% of the handouts. The limited number of lessons and handouts received by mothers may be the result of mothers not receiving enough visits to cover the 24 lesson HEALTH curriculum. We found on average, home visitors reported 14 visits with a mother. Even though the number of visits may reduce the number of opportunities to deliver HEALTH lessons, still only 26% of mothers received the small number of eight core HEALTH lessons. It is important to note the only data available is from a simple metric of how many lessons are delivered. This measure does not capture use of HEALTH in terms of a mother getting the lessons that are most relevant and in line with what she needs. Though we found usage may be higher in certain groups (mothers with obesity, not Hispanic, making, those reporting an annual household income of $30,000 or more), usage overall was very low. Comparison to previous research is limited by the heterogeneity of EBIs and study types but results on usage seem to be lower than other studies [57, 62]. One home visiting program for parents, though much shorter in duration, found 97% of the intervention elements were completed and 71% of participants received all ten sessions [63]. The original HEALTH study assessed content delivered; however, comparisons are less meaningful due to differences in intervention delivery (research staff in the original study compared to PAT providers in this study) and no set number of required HEALTH lessons/visits [64, 65].

The limited usage of HEALTH could be due to the impact of COVID-19 on PAT retention and mother engagement. While home visiting was quick to transition to virtual visits [40], providing much needed services during the pandemic, home visiting models and studies conducting research in home visiting saw decreases in participation [38–40, 52, 54]. Ensuring mothers have sufficient visits to receive HEALTH and challenges for retention are not unique to the pandemic and should be addressed. Implementation strategies which support accessibility and acceptability such as facilitating peer learning, creating program guides, building partner relationships, meeting community partner needs, and choosing strategic partners can also support usage and ensure mothers receive more of the HEALTH content [30, 31]. Facilitating peer learning provides networking and collaborating opportunities for home visitors to share their experiences delivering HEALTH in order to learn from each other. Creating program guides can help home visitors implement HEALTH in a way that supports a greater number of lessons and more HEALTH content being provided to mothers. Building partner relationships, meeting community partner needs, and choosing strategic partners, which focus on social needs linkages, may help address families’ needs more effectively, so home visitors can focus on HEALTH delivery during visits, increasing HEALTH usage. While provider training was used as an implementation strategy in the HEALTH D&I study, this strategy could be expanded on to include training supervisors on how to best support home visitors in delivering HEALTH content.

### Limitations and strengths

This study has limitations that are important to note. First, as mentioned previously in the discussion section, the measure for accessibility (home visitor referral to the HEALTH D&I study) may not fully reflect choice to adopt HEALTH in a real-world context. HEALTH D&I eligibility criteria around weight and pregnancy may have influenced which mothers and how many a home visitor chose to refer to HEALTH (e.g., a home visitor may have avoided referring mothers they knew were pregnant). Another limitation is the sample size and the inability to statistically compare groups of mothers across aspects of coverage. The HEALTH D&I study took place during COVID-19, which influenced recruitment and retention rates. We relied on home visitor report to determine if visits happened and what occurred during those visits. If home visitors did not complete a visit record, that visit could not be included in the analysis; this results in an underestimation of the visits families received. Further, if home visitors were more likely to report visits where they did cover HEALTH content, we may have overestimated the extent to which HEALTH was covered as a proportion of visits. This study also has strengths such as expanding on RE-AIM with the Shengelia et al. coverage framework to inform conceptualization and measurement of community-based EBI implementation outcomes and prospective assessment of implementation. Further, this study describes coverage for a community-based EBI and identifies potential implementation strategies for addressing coverage gaps and building on coverage strengths.

## Conclusion

We used D&I frameworks with the Shengelia et al. Coverage framework to inform coverage of the community-based EBI HEALTH during COVID-19. Using the HEALTH D&I study data we found HEALTH was highly acceptable among mothers but limited in accessibility and usage. These findings suggest HEALTH has the potential to engage mothers even in the context of COVID-19. Implementation strategies specific to community settings such as choosing strategic partner organizations, building partner relationships, and meeting partner needs may help ensure HEALTH is accessible to mothers and enough HEALTH content is delivered. Describing indicators of HEALTH implementation during the pandemic provides insight for translation of community-based EBIs to support EBI adoption and implementation in community settings and offers insights into home visiting service delivery during COVID-19.

## Data Availability

The datasets used and/or analysed during the current study are available from the corresponding author on reasonable request.

## Abbreviations

EBI: Evidence-based intervention
PAT: Parents as Teachers
HEALTH: Healthy Eating and Active Living Taught at Home
